# Rucaparib in recurrent ovarian cancer: real-world experience from the rucaparib early access programme in Spain – A GEICO study

**DOI:** 10.1186/s12885-022-10191-5

**Published:** 2022-11-08

**Authors:** Alfonso Yubero, Aranzazu Barquín, Purificación Estévez, Bella Pajares, Luisa Sánchez, Piedad Reche, Jesús Alarcón, Julia Calzas, Lydia Gaba, José Fuentes, Ana Santaballa, Carmen Salvador, Luis Manso, Ana Herrero, Álvaro Taus, Raúl Márquez, Julia Madani, María Merino, Gloria Marquina, Victoria Casado, Manuel Constenla, María Gutiérrez, Alba Dosil, Antonio González-Martín

**Affiliations:** 1 Medical Oncology, Hospital Clínico Universitario Lozano Blesa, Zaragoza, Spain; 2 Medical Oncology, Centro Integral Oncológico Clara Campal, Madrid, Spain; 3 Medical Oncology, Hospital Universitario Virgen del Rocío, Sevilla, Spain; 4 Medical Oncology, Hospital Universitario Virgen de la Victoria, Málaga, Spain; 5 Medical Oncology, Clínica Universidad de Navarra, Madrid, Spain; 6 Medical Oncology, Hospital Universitario Torrecárdenas, Almería, Spain; 7 Medical Oncology, Hospital Universitario Son Espases, Mallorca, Spain; 8 Medical Oncology, Hospital Universitario de Fuenlabrada, Madrid, Spain; 9 Department of Medical Oncology, Translational Genomics and Targeted Therapeutics in Solid Tumors, Hospital Clínic de Barcelona, Institut D’Investigacions Biomèdiques August Pi I Sunyer (Idibaps), Barcelona, Spain; 10 Medical Oncology, Hospital Universitario Virgen de Valme, Sevilla, Spain; 11 Medical Oncology, Hospital Universitario i Politècnic la Fe, Valencia, Spain; 12 Medical Oncology, Hospital Lluís Alcanyís de Xàtiva, Xátiva, Spain; 13 Medical Oncology, Hospital Universitario 12 de Octubre, Madrid, Spain; 14 Medical Oncology, Hospital Universitario Miguel Servet, Zaragoza, Spain; 15 Medical Oncology, Hospital del Mar, Barcelona, Spain; 16 Medical Oncology, MD Anderson Cancer Center, Madrid, Spain; 17 Medical Oncology, Hospital Universitario San Jorge, Huesca, Spain; 18 Medical Oncology, Hospital Universitario Infanta Sofía, Madrid, Spain; 19 Medical Oncology, Hospital Clínico San Carlos, Madrid, Spain; 20 Medical Oncology, Fundación Jiménez Díaz, Madrid, Spain; 21 Medical Oncology, Complejo Hospitalario Universitario de Pontevedra, Pontevedra, Spain; 22 Medical Oncology, Hospital Universitario Txagorritxu, Araba, Spain; 23 Clovis Oncology , Madrid, Spain; 24 Centre for Applied Medical Research, Pamplona, Spain

**Keywords:** Rucaparib, Recurrent ovarian cancer, Maintenance, Treatment, PARP inhibitor

## Abstract

**Background:**

Rucaparib is a poly(ADP-ribose) polymerase inhibitor approved in Europe as maintenance therapy for recurrent platinum-sensitive (Pt-S) ovarian cancer (OC). The Rucaparib Access Programme (RAP) was designed to provide early access to rucaparib for the above-mentioned indication, as well as for patients with *BRCA*-mutated Pt-S or platinum-resistant (Pt-R) OC and no therapeutic alternatives.

**Methods:**

In this observational, retrospective study we analysed the efficacy and safety of rucaparib within the RAP in Spain. Hospitals associated with the Spanish Ovarian Cancer Research Group (GEICO) recruited patients with high-grade epithelial ovarian, fallopian tube, or primary peritoneal cancer treated with rucaparib 600 mg twice daily as maintenance or treatment (Pt-S/Pt-R) in the RAP. Baseline characteristics, efficacy, and safety data were collected.

**Results:**

Between July 2020 and February 2021, 51 patients treated in 22 hospitals in the RAP were included in the study. Eighteen patients with a median of 3 (range, 1–6) prior treatment lines received rucaparib as maintenance; median progression-free survival (PFS) for this group was 9.1 months (95% confidence interval [CI], 4.2–11.6 months). Among 33 patients (median 5 [range, 1–9] prior treatment lines) who received rucaparib as treatment, 7 and 26 patients had Pt-S and Pt-R disease, respectively. Median PFS was 10.6 months (95% CI, 2.5 months-not reached) in the Pt-S group and 2.2 months (95% CI, 1.1–3.2 months) in the Pt-R group. Grade ≥ 3 treatment-emergent adverse events were reported in 39% of all patients, the most common being anaemia (12% and 15% in the maintenance and treatment groups, respectively). At data cut-off, 5 patients remained on treatment.

**Conclusion:**

Efficacy results in these heavily pre-treated patients were similar to those from previous trials. The safety profile of rucaparib in real life was predictable and manageable.

## Background

Ovarian cancer (OC) is the third most common gynaecological tumour and was the second leading cause of death from gynaecological cancer in women worldwide in 2020 [1]. Despite optimal surgery and platinum-based treatment, disease will relapse in approximately 80–85% of patients with advanced OC. Poly(ADP-ribose) polymerase (PARP) inhibitors (PARPis) have changed the landscape of advanced OC. Based on their efficacy and tolerability, these agents have been rapidly incorporated into the treatment algorithm. Currently, efforts in recurrent OC management are focused on maintenance treatment with anti-angiogenic drugs and PARPis [2, 3].

Rucaparib is an oral, small molecule inhibitor of PARP-1/2/3 that has shown preclinical and clinical activity in epithelial OC [4–6]. Rucaparib was approved by the European Medicines Agency (EMA) in January 2019 as maintenance for patients with recurrent platinum-sensitive (Pt-S) OC who have a complete or partial response (CR or PR) to platinum-based chemotherapy [7]. Rucaparib approval was based on the results of the ARIEL3 (NCT01968213) clinical trial, in which rucaparib significantly improved progression-free survival (PFS) over placebo across the three predefined cohorts based on genomic characteristics, regardless of the genetic background or biomarker status of the patients [5]. In Study 10 (NCT01482715), ARIEL2 (NCT01891344) and ARIEL4 (NCT02855944) [6, 8, 9], rucaparib demonstrated improved PFS in the treatment setting in patients with *BRCA*-mutated recurrent Pt-S or platinum-resistant (Pt-R) OC who had received two or more prior platinum chemotherapy regimens.

In March 2018, Clovis Oncology initiated the Rucaparib Access Programme (RAP) in Europe, an early access programme for the licensed indication. In addition, the RAP was designed to provide rucaparib for off-label use in patients with *BRCA*-mutated Pt-S or Pt-R OC and no other therapeutic options [10]. The RAP has been active in Spain since September 2018 and was closed to new patients in March 2020. Overall, 60 patients were treated in the RAP in Spain.

Here, we present results from a retrospective study conducted by the Spanish Ovarian Cancer Research Group (GEICO) in patients with recurrent OC treated with rucaparib within the RAP in Spain. The aim of the study was to understand better the management of rucaparib in a real-life setting in an unselected population, to optimise its future use.

## Methods

### Study design and patients

In this multicentre, retrospective, observational study, 22 GEICO-associated hospitals recruited patients treated with rucaparib within the RAP in Spain since September 2018. The study protocol was approved by the ethics committees of the participating sites and performed according to the Declaration of Helsinki and local laws and regulations. Eligible patients were adult women (≥ 18 years at diagnosis) with high-grade epithelial ovarian, fallopian tube, or primary peritoneal cancer who had received at least one dose of rucaparib within the RAP. The starting dose of rucaparib was 600 mg twice daily; rucaparib could have been administered as maintenance for patients with recurrent Pt-S OC or as treatment for patients with Pt-S or Pt-R recurrent OC and a *BRCA* mutation. Accessible patients provided written informed consent. In accordance with Spanish laws, informed consent was not required from inaccessible patients.

### Data collection and outcomes

Patient characteristics, dosing, efficacy, and safety data were collected and analysed. Patient characteristics included age, histology, mutational status of *BRCA* and other homologous recombination repair genes, previous relapses, previous treatment, and treatment-free interval. Rucaparib dosing data included starting dose, dose interruptions, dose reductions, treatment discontinuations, and duration of treatment. Safety data comprised all haematological and non-haematological adverse events related to rucaparib that were available in the medical records, graded according to Common Terminology Criteria for Adverse Events, version 5.0. Main efficacy parameters were investigator-assessed radiological best response by Response Evaluation Criteria In Solid Tumours (RECIST) version 1.1, biological best response by Rustin criteria, duration of response, and investigator-assessed PFS. Patient data were extracted from source medical records available at the participating sites and entered on a web-based electronic case report form system.

### Statistical methods

The study population included all available patients from the participating sites who received at least one dose of rucaparib within the RAP. The initially estimated number of participants was based on the accrual rate of the RAP until rucaparib became available in Spain. There was no formal sample size calculation. PFS was estimated using Kaplan–Meier methodology and medians were reported with associated 95% confidence intervals (CIs). All analyses were descriptive, performed in the overall population and in subgroups according to therapy setting (maintenance or treatment). In addition, PFS was analysed according to platinum sensitivity in the treatment subgroup (Pt-S or Pt-R). Descriptive subgroup analyses according to age at start of rucaparib treatment (< 70 vs. ≥ 70 years) were prespecified. Associations between variables were tested by conventional statistical analysis (Pearson and/or Spearman tests). The World Programming System (WPS) platform (SAS language software) was used for all statistical analyses.

## Results

Between July 2020 and February 2021, 51 of the 60 patients treated in the RAP were included in the study. The data cut-off date was 31st March, 2021. Eighteen patients received rucaparib as maintenance after response to platinum-based chemotherapy and 33 patients were treated with rucaparib monotherapy following progression on the previous treatment line; of these, 26 patients had Pt-R disease and 7 had Pt-S disease. Baseline characteristics of all patients are shown in Table 1.

**Table 1:** Patient populations and baseline characteristics

Characteristic	Maintenance (***n*** = 18)	Treatment (***n*** = 33)	Total (***n*** = 51)
**Age**, years	65.5 (44–86)	63 (36–86)	63 (36–86)
**Diagnosis**			
Epithelial ovarian cancer	17 (94%)	30 (91%)	47 (92%)
Fallopian tube or primary peritoneal cancer	1 (6%)	3 (9%)	4 (8%)
**Histology**			
Serous	18 (100%)	31 (94%)	49 (96%)
Other^*^	0	2 (6%)	2 (4%)
**FIGO stage**			
I/II	2 (11%)	2 (6%)	4 (8%)
III	15 (83%)	25 (76%)	40 (78%)
IV	1 (6%)	5 (15%)	6 (12%)
Unknown	0	1 (3%)	1 (2%)
**ECOG PS**			
0	7 (39%)	12 (36%)	19 (37%)
1	10 (56%)	15 (45%)	25 (49%)
2	0	3 (9%)	3 (6%)
Unknown	1 (6%)	3 (9%)	4 (8%)
***BRCA*** **status**			
*BRCA* mutant	3 (17%)	28 (85%)	31 (61%)
Germline^†^	2 (11%)	21 (64%)	23 (45%)
Somatic^†^	1 (6%)	8 (24%)	9 (18%)
*BRCA* wildtype	13 (72%)	3 (9%)	16 (31%)
Unknown	2 (11%)	2 (6%)	4 (8%)
**Mutation in other HRR genes**			
*RAD51C*	1 (6%)	1 (3%)	2 (4%)
**Primary cytoreductive surgery**			
PDS	12 (67%)	23 (70%)	35 (69%)
IDS	5 (28%)	10 (30%)	15 (29%)
No surgery	1 (6%)	0	1 (2%)
**Primary surgery outcome** ^**‡**^			
R0	9 (53%)	22 (67%)	31 (62%)
R1	8 (47%)	9 (27%)	17 (34%)
Unknown	0	2 (6%)	2 (4%)
**Salvage surgery**			
Yes	6 (35%)	6 (18%)	12 (24%)
No	11 (65%)	27 (82%)	38 (76%)
**Number of previous treatment lines**	3 (1–6)	5 (1–9)	4 (1–9)
1	2 (11%)	2 (6%)	4 (8%)
2	5 (28%)	3 (9%)	8 (16%)
3	6 (33%)	5 (15%)	11 (22%)
4	2 (11%)	5 (15%)	7 (14%)
≥ 5	3 (17%)	18 (55%)	21 (41%)
**Previous bevacizumab use**	10 (56%)	17 (52%)	27 (53%)
**Prior PARPi**	1 (6%)	13 (39%)	14 (27%)
**Platinum status**			
Platinum resistant	NA	26 (79%)	26 (51%)
Platinum sensitive	18 (100%)	7 (21%)	25 (49%)
**Measurable disease** (investigator assessed)			
Yes	9 (50%)	28 (85%)	37 (73%)
No	9 (50%)	5 (15%)	14 (27%)
Unknown	0	0	0
**Response to last platinum (RECIST)**			
CR	6 (33%)	NA	NA
PR	10 (56%)	NA	NA
SD	2 (11%)	NA	NA
**Comorbidities** ^§^			
Hypertension	4 (22%)	6 (18%)	10 (20%)
Diabetes mellitus	2 (11%)	1 (3%)	3 (6%)
Obesity	2 (11%)	0	2 (4%)
Hypothyroidism	0	2 (6%)	2 (4%)

Data are median (range) or n (%). CR: complete response; ECOG PS: Eastern Cooperative Oncology Group performance status; FIGO: International Federation of Gynecology and Obstetrics; HRR: homologous recombination repair; IDS: interval debulking surgery. NA: not applicable; PDS: primary debulking surgery. PR: partial response. ^*^Endometrioid and clear-cell histology. ^†^1 patient had both germline and somatic *BRCA1* mutations. ^‡^1 patient from the maintenance subgroup did not undergo surgery.^**§**^Most frequent comorbidities (reported in at least 2 patients)

In the maintenance group, median age was 65.5 (range 44–86) years and 94% of patients were diagnosed with epithelial OC. 72% of patients had *BRCA-*wildtype tumours and 1 patient (6%) had a *RAD51C* mutation. The median number of previous treatment lines was 3 (range 1–6) and 61% of patients had received ≥ 3 previous lines. Eastern Cooperative Oncology Group performance status (ECOG PS) was 1 in 56% patients, 56% of patients had achieved a PR to prior platinum-based chemotherapy and 50% of patients presented with measurable disease.

In the treatment group, median age was 63 (range 36–86) years and 91% of patients were diagnosed with epithelial OC. In 85% of patients, tumours harboured a *BRCA* mutation, and 1 patient (3%) had a *RAD51C* mutation. The median number of previous lines was 5 (range 1–9) and 55% of the patients had received ≥ 5 previous lines. Before initiating rucaparib, 45% and 9% of patients had ECOG PS 1 and 2, respectively, and 85% of patients presented with measurable disease.

Overall, 53% of the patients had received previous bevacizumab and 14 patients (27%) had received a prior PARPi. Of those, 1 patient received rucaparib as maintenance, 1 patient as treatment for Pt-S disease and 12 patients as treatment for Pt-R disease.

**Fig. 1:**
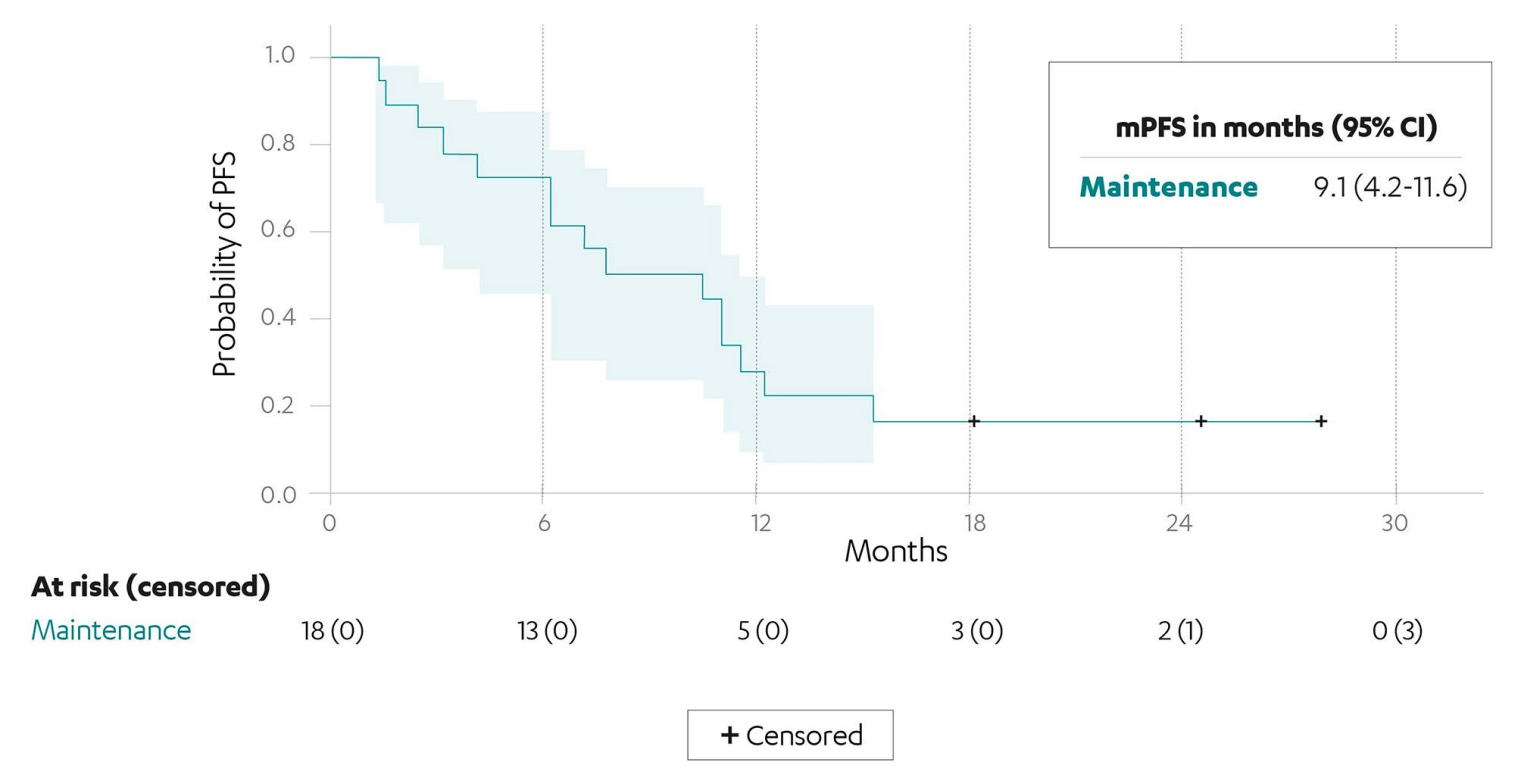
Investigator-assessed PFS in the maintenance subgroup (*n* = 18). CI: confidence interval; mPFS: median progression-free survival; +: censored patients

**Fig. 2:**
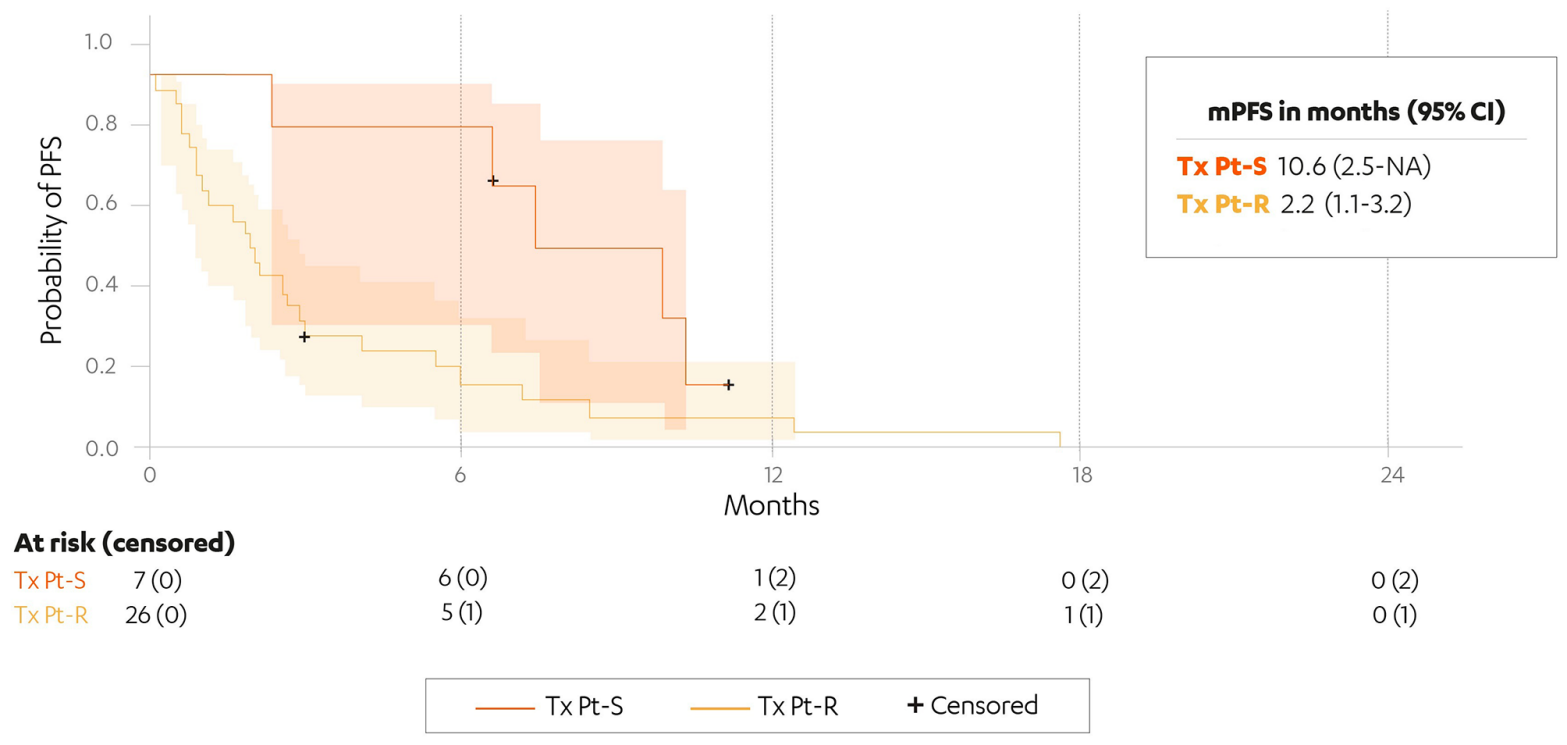
Investigator-assessed PFS in the Pt-S treatment subgroup (*n* = 7) and the Pt-R treatment subgroup (*n* = 26). CI: confidence interval; mPFS: median progression-free survival; Tx Pt-S: treatment, platinum-sensitive disease; Tx Pt-R: treatment, platinum-resistant disease; +: censored patients

Median PFS (mPFS) in the maintenance group was 9.1 months (95% CI, 4.2–11.6) (Fig. 1). mPFS in the Pt-S and Pt-R treatment groups was 10.6 (95% CI, 2.5-not reached) and 2.2 (95% CI, 1.1–3.2) months, respectively (Fig. 2).

In the treatment group, 19 of 28 patients with measurable disease at baseline were radiologically evaluable: 4 patients with Pt-S disease and 15 patients with Pt-R disease. Nine patients were not assessable for response. In the Pt-S subgroup, 1 patient achieved a PR and 2 patients had stable disease (SD) as best response to rucaparib. Among evaluable patients with Pt-R disease, the disease control rate (CR, PR or SD) was 33% (Table 2).

**Table 2:** Radiological and biological best overall response in patients with measurable disease at baseline (treatment group)

	Pt-S (***n*** = 4)	Pt-R (***n*** = 24)	Total (***n*** = 28)
**Radiological Best Overall Response**			
Investigator-assessed RECIST ORR	1 (25%)	3 (13%)	4 (14%)
Complete response	0	0	0
Partial response	1 (25%)	3 (13%)	4 (14%)
Stable disease	2 (50%)	2 (8%)	4 (14%)
Progressive disease	1 (25%)	10 (42%)	11 (39%)
Not assessable	0	9 (38%)	9 (32%)
**Biological best overall response**			
Response	1 (25%)	2 (8%)	3 (11%)
Stabilisation	1 (25%)	6 (25%)	7 (25%)
Progression	1 (25%)	5 (21%)	6 (21%)
Not assessable	1 (25%)	11 (46%)	12 (43%)

Data are n (%). ORR: objective response rate. Pt-S: platinum-sensitive disease. Pt-R: platinum-resistant disease. RECIST: Response Evaluation Criteria In Solid Tumors version 1.1

Overall, treatment-emergent adverse events (TEAEs) of any grade were reported in 44 patients (86%) patients. Grade ≥ 3 TEAEs occurred in 39% of patients.

In the maintenance group, a TEAE of any grade occurred in 89% of patients. The most common TEAEs of any grade (reported in at least 25% patients) were nausea, alanine aminotransferase/aspartate aminotransferase increase and fatigue. Grade 3 toxicities were reported in 4 patients (22%) and there were no grade 4 TEAEs. The most common grade 3 TEAE was anaemia (11%). No grade ≥ 3 thrombocytopenia or neutropenia was observed (Table 3 A).

In the treatment group, 85% of patients had a TEAE, the most common being anaemia and thrombocytopenia. Grade ≥ 3 TEAEs were reported in 16 patients (49%), the most common being anaemia (15%). The remaining grade ≥ 3 TEAEs were reported in ≤ 2 patients (Table 3B).

**Table 3:** Most common TEAEs (reported at any grade in ≥ 2 patients) for maintenance and treatment

	ALL GRADES	GRADE 3
**Maintenance (** ***n*** **= 18)**		
Nausea	7 (39%)	1 (6%)
ALT/AST increase	5 (28%)	1 (6%)
Fatigue	5 (28%)	0
Diarrhoea	4 (22%)	0
Creatinine increased	4 (22%)	0
ALP increase	2 (11%)	1 (6%)
Anaemia	2 (11%)	2 (11%)
Neutropenia	2 (11%)	0
Dysgeusia	2 (11%)	0
Vomiting	2 (11%)	0
Abdominal pain	2 (11%)	0
Anorexia	2 (11%)	0
**Treatment (n=33)**		
Anaemia	13 (39%)	5 (15%)
Thrombocytopenia	10 (30%)	2 (6%)
Fatigue	7 (21%)	2 (6%)
Nausea	5 (15%)	0
ALT/AST increase	4 (12%)	0
Neutropenia	3 (9%)	2 (6%)
Vomiting	3 (9%)	2 (6%)
ALP increase	3 (9%)	1 (3%)
Asthenia	3 (9%)	0
Abdominal pain	2 (6%)	1 (3%)
Hyporexia	2 (6%)	0
Constipation	2 (6%)	0

Data are n (%). TEAEs were graded according to Common Terminology Criteria for Adverse Events version 5.0, with the highest grade reported if patients reported the same event at more than one grade. ALP: alkaline phosphatase. ALT: alanine aminotransferase. AST: aspartate aminotransferase. TEAE: treatment-emergent adverse event.

Myelodysplastic syndrome (MDS) was reported in 1 > 70-year-old patient who received rucaparib as fourth-line treatment for Pt-R OC for more than 16 months.

All patients in the maintenance group began rucaparib at the recommended dose of 600 mg twice daily. The median treatment duration was 7.5 (range 1.1–15.5) months and 20% of patients continued treatment for > 12 months. Dose interruptions and reductions were reported in 56% and 61% of patients, respectively. Only 1 patient discontinued rucaparib maintenance due to toxicity, and at the data cut-off date, 3 patients remained on treatment.

In the treatment group, 91% began rucaparib at the recommended starting dose of 600 mg twice daily. The median treatment duration was 8.6 (range 3–12) and 2.1 (range 0–16) months for patients with Pt-S and Pt-R disease, respectively. Almost all (97%) had a treatment duration < 12 months. Treatment interruptions and dose reductions occurred in 63% and 44% of patients, respectively. Four patients discontinued rucaparib due to toxicity, and at the data cut-off date, 2 patients remained on treatment (Table 4).

**Table 4:** Treatment exposure

	Maintenance (***n*** = 18)	Treatment (***n*** = 33)	Total (***n*** = 51)
**Initial dose 600 mg twice daily**	18 (100%) ​	30 (91%) ​	48 (94%)
**Treatment duration**, months	7.5 (1.1–15.5) ​	2.4 (0.2–16.7) ​	3.3 (0.2–16.7) ​
Treatment duration*			
0–12 months	12 (80%)	30 (97%)	42 (91%)
12–24 months	3 (20%)	1 (3%)	4 (9%)
**Dose interruptions** ^**†**^	10 (56%)	20 (63%)	30 (60%)
0	8 (44%)	12 (38%)	20 (40%)
1	8 (44%)	13 (41%)	21 (42%)
2	0	7 (22%)	7 (14%)
≥ 3	2 (11%)	0	2 (4%)
**Dose reductions** ^**†**^	11 (61%)	14 (44%)	25 **(**50%)
0	7 (39%)	18 (56%)	25 (50%)
1	5 (28%)	11 (34%)	16 (32%)
2	5 (28%)	3 (9.4)	8 (16%)
3	1 (6%)	0	1 (2%)
**Reason for discontinuation**			
Progression	13 (72%)	25 (76%)	38 (75%)
Toxicity​	1 (6%)	4 (12%)	5 (10%)
Other^‡^	1 (6%)	2 (6%)	3 (6%)
**On treatment**	3 (17%)	2 (6%)	5 (10%)

Data are median (range) or n (%). *Percentages calculated based on patients who had discontinued treatment. ^†^1 patient in the treatment group was excluded from the analysis as the recommended dose modification scheme was not followed. ^‡^Doctor’s or patient’s decision.

In subgroup analyses according to age < 70 vs. ≥ 70 years, the incidence of any-grade TEAEs was similar between subgroups (85% vs. 90%, respectively); however, grade ≥ 3 TEAEs were more common in the older subgroup (50%, vs. 34% in younger patients). Dose interruptions and reductions of rucaparib were also more frequent in the older subgroup (Table 5).

**Table 5:** Safety and treatment exposure according to age

Parameter	< 70 years (***n*** = 41)	≥ 70 years (***n*** = 10)
**Any grade TEAE**	35 (85%)	9 (90%)
**Grade ≥ 3 TEAE**	14 (34%)	5 (50%)
**Dose interruptions** ^*^		
Yes	23 (56%)	7 (78%)
No	18 (44%)	2 (22%)
**Dose reduction** ^*^		
Yes	18 (44%)	7 (78%)
No	23 (56%)	2 (22%)
**Reason for discontinuation**		
Progression	32 (78%)	6 (60%)
Toxicity	3 (7%)	2 (20%)
Others^**†**^	3 (7%)	0
**On treatment**	3 (7%)	2 (20%)

Data are n (%). *1 patient aged > 70 years was excluded from the analysis as the recommended dose modification scheme was not followed. †Doctor’s or patient’s decision.

## Discussion

This GEICO retrospective study evaluated the efficacy and tolerability of rucaparib in patients with recurrent OC treated in a real-world setting. The use of rucaparib in real-life, either as maintenance or treatment for recurrent OC, showed a favourable benefit-risk profile outside clinical trials in Spain.

The results from this retrospective study appear similar to previous data reported for rucaparib in pivotal clinical trials. However, the interpretation of PFS results from our study may be challenging due to the heterogeneity of the patient population and the nature of the study. For that reason, indirect comparisons using these data and any conclusions drawn from them ought to be interpreted with caution. Considering all these caveats, our results show efficacy of rucaparib in recurrent OC. In this study, the mPFS in the maintenance group was 9.1 months, while the mPFS in ARIEL3 was 10.8 months in the intention-to-treat cohort. Although slightly inferior, our results display similar PFS to ARIEL3 and the minor discordance in the median values could be explained by differences in clinical factors between the 2 populations, including *BRCA* mutation status, number of previous treatment lines, and measurable disease at baseline. Compared with ARIEL3, our study included fewer patients with *BRCA*-mutated tumours (17% vs. 35%) and *BRCA* status was unknown in 11% of the patients. In addition, the maintenance group included a more heavily pretreated population (median number of previous lines: 3 [range, 1–6] in this study vs. 2 [range, 2–3] in ARIEL3) and more patients had measurable disease at baseline (50% vs. 38%) [5].

mPFS with rucaparib as treatment for Pt-S OC was 10.6 months, similar to the ARIEL4 clinical trial. In ARIEL4, patients were classified based on their platinum sensitivity status: partially Pt-S (platinum-free interval [PFI] ≥ 6–12 months), fully Pt-S (PFI ≥ 12 months) and Pt-R (PFI < 6 months). ARIEL4 results showed mPFS of 12.9 and 8.0 months in patients with fully and partially Pt-S OC, respectively. In our study, the criteria to define platinum sensitivity were simplified: patients were considered to have Pt-S disease if PFI was ≥ 6 months and Pt-R disease if PFI was < 6 months. The treatment group of our study was enriched with Pt-R disease and the mPFS in this subgroup was 2.2 months, shorter than the mPFS of 6.4 months in ARIEL4 for this population. Again, some clinical factors may explain the differences. Patients treated with rucaparib in the Pt-R setting in this study represented a heavily pretreated population (median 5 previous lines [range 2–9]), and 12 of 26 patients had received a prior PARPi before rucaparib. Prior PARPi was an exclusion criterion in ARIEL4. Overall, our treatment population was less fit (9% of patients in the treatment group had ECOG PS 2 whereas patients with ECOG PS 2 were not eligible for enrolment in ARIEL4) [9]. In addition, *BRCA* status was wild-type or unknown in 5 patients who received rucaparib as treatment.

The overall safety profile of rucaparib in this real-life setting was acceptable and consistent with previously reported data. In our study, no new safety signals were identified, and generally, we reported a lower incidence of grade ≥ 3 TEAEs. In the maintenance group, any-grade anaemia was less frequent than in ARIEL3 (11% vs. 39%, respectively) and importantly, grade 3/4 anaemia was only half as common in our study (11% vs. 22%, respectively). Notably, no grade 3/4 neutropenia or thrombocytopenia was reported in the maintenance group (vs. 8% and 5%, respectively in ARIEL3). Regarding non-haematological toxicities, any-grade nausea and fatigue were also less frequent in our study than in ARIEL3 (nausea: 39% vs. 76%, respectively; fatigue: 28% vs. 71%) [11]. Finally, across the entire study population, the incidence of MDS was low (1 patient with Pt-R OC), as reported previously with PARPis [5, 9, 12, 13].

The proportions of patients requiring dose reductions or treatment interruptions were similar to ARIEL3 (in the maintenance group), and to the integrated analysis of Study 10 and ARIEL2, and ARIEL4 (in the treatment group). Furthermore, the discontinuation rate was lower than in pivotal clinical trials and was caused mainly by disease progression [8, 9, 11]. As of 22nd September 2022, 4 patients remain on treatment: to date, 3 patients have been on rucaparib maintenance for 3.5–4 years and 1 patient with Pt-S OC has been on rucaparib treatment for 4 years. 1 patient with Pt-S OC discontinued rucaparib treatment due to progression after the data cut-off date.

There are some obvious limitations to our analysis, mostly inherent to the nature of non-interventional real-world studies. The patient population was heterogenous, and therapeutic decisions and patient evaluations were at the physician’s discretion and thus may not be homogenous between sites. Differences in local testing for *BRCA* germline or somatic mutations could also be considered a limitation. Furthermore, data were collected retrospectively, and therefore, may be biased. Importantly, the number of patients was smaller than in the above-mentioned clinical trials. However, despite these limitations, rucaparib given as maintenance and treatment, even in an unselected and heavily pretreated population, showed a consistent efficacy and safety profile in a real-life setting.

The efficacy of PARPis as maintenance for recurrent OC has been reported in randomised clinical trials. However, there is a need for data validation in routine clinical practice treating unselected, heterogenous and, potentially, less fit patients. Few studies have evaluated real-world evidence with PARPis. The Italian MITO working group reported the results of the largest real-world study in *BRCA*-mutated patients treated with olaparib. As in our study, efficacy and safety observed in real-life setting for olaparib was similar to randomised clinical trials [14]. Additional smaller series with shorter follow-up have been reported for olaparib [15, 16]. Regarding niraparib, real-world data are more sparse. A retrospective study in a real-life setting analysed niraparib safety at a starting dose of 200 mg/day in patients from the USA, but efficacy was not assessed in this study [17].

This GEICO study is, to our knowledge, the largest to report efficacy and safety data of rucaparib as maintenance and treatment of recurrent OC in a real-life setting. The results of our study not only confirm the activity of rucaparib maintenance and treatment in a real-world population of patients with recurrent OC, but also show a predictable and manageable safety profile.

Real-world data with PARPis in both the front-line and the recurrent setting are still scarce and further research is warranted. Unravelling the best biomarkers of response, establishing the optimal treatment sequence for each patient, and understanding the scenario after treatment with a prior PARPi will be crucial in the future of recurrent OC management.

## Conclusion

This study represents real-world evidence of patients treated with rucaparib outside clinical trials in Spain. Efficacy results of rucaparib, even in heavily pre-treated patients, are similar to those from pivotal clinical trials. The safety profile of rucaparib in a real-life setting is manageable and predictable.

## Data Availability

The datasets generated and/or analysed during the current study are not publicly available due to data protection laws but are available from the corresponding author on reasonable request and with permission from GEICO.

## List of abbreviations

ALP: alkaline phosphatase
ALT: alanine aminotransferase
AST: aspartate aminotransferase
CI: confidence interval
CR: complete response
ECOG PS: Eastern Cooperative Oncology Group performance status
EMA: European Medicines Agency
FIGO: International Federation of Gynecology and Obstetrics
GEICO: Spanish Ovarian Cancer Research Group
HRR: homologous recombination repair
IDS: interval debulking surgery
MDS: myelodysplastic syndrome
mPFS: median progression-free survival
NA: not applicable
NR: not reached
OC: ovarian cancer
ORR: objective response rate
PARP: poly(ADP-ribose) polymerase
PARPi: PARP inhibitor
PDS: primary debulking surgery
PFI: platinum-free interval
PFS: progression-free survival
PR: partial response
Pt-R: platinum-resistant
Pt-S: platinum-sensitive
RAP: rucaparib access programme
RECIST: Response Evaluation Criteria In Solid Tumors
SD: stable disease
TEAEs: treatment-emergent adverse events
Tx: treatment
